# Incubation of MDCO-216 (ApoA-IMilano/POPC) with Human Serum Potentiates ABCA1-Mediated Cholesterol Efflux Capacity, Generates New Prebeta-1 HDL, and Causes an Increase in HDL Size

**DOI:** 10.1155/2014/923903

**Published:** 2014-11-12

**Authors:** Herman J. Kempen, Dorota B. Schranz, Bela F. Asztalos, James Otvos, Elias Jeyarajah, Denise Drazul-Schrader, Heidi L. Collins, Steven J. Adelman, Peter L. J. Wijngaard

**Affiliations:** ^1^The Medicines Company (Schweiz) GmbH, Talstrasse 59, 8002 Zürich, Switzerland; ^2^Pacific Biomarkers LLC, 645 Elliott Avenue West, Suite 300, Seattle, WA 98119, USA; ^3^Lipid Metabolism Laboratory, Tufts University, 711 Washington Street, Boston, MA 02111, USA; ^4^Liposcience LLC, 2500 Sumner Boulevard, Raleigh, NC 27616, USA; ^5^Vascular Strategies LLC, 5110 Campus Drive, Suite 150, Plymouth Meeting, PA 19462, USA

## Abstract

MDCO-216 is a complex of dimeric ApoA-IMilano and palmitoyl oleoyl phosphatidylcholine (POPC), previously shown to reduce atherosclerotic plaque burden. Here we studied the effect of incubation of human plasma or serum with MDCO-216 on cholesterol efflux capacity from J774 cells, on prebeta-1 high density lipoprotein (prebeta-1 HDL) and on HDL size assessed by proton nuclear magnetic resonance (^1^H-NMR). MDCO-216 incubated in buffer containing 4% human serum albumin stimulated both ABCA1-mediated efflux and ABCA1-independent cholesterol efflux from J774 macrophages. When incubated with human serum a dose- and time-dependent synergistic increase of the ABCA1-mediated efflux capacity were observed. Using a commercially available ELISA for prebeta-1 HDL, MDCO-216 as such was poorly detected (12–15% of nominal amount of protein). Prebeta-1 HDL was rapidly lost when human plasma alone is incubated at 37°C. In contrast, incubation of human plasma with MDCO-216 at 37°C produced a large amount of new prebeta-1 HDL. Native 2D electrophoresis followed by immunoblotting with an apoA-I antibody, which also detects ApoA-I Milano, confirmed the increase in prebeta-1 HDL upon incubation at 37°C. With the increase of prebeta-1 HDL, the concomitant disappearance of the small alpha-3 and alpha-4 HDL and MDCO-216 and an increase in the large alpha-1 and alpha-2 HDL were observed. Immunoblotting with Mab 17F3 specific for ApoA-I Milano showed the appearance of ApoA-I Milano in alpha-1 and alpha-2, but not in prebeta-1 HDL. ^1^H-NMR analysis of plasma incubated with MDCO-216 confirmed rapid disappearance of small-sized HDL particles and increase of medium- and large-sized HDL particles accompanied with a decrease in total HDL particle number. In conclusion, incubation of human plasma or serum with MDCO-216 strongly enhanced ABCA1-mediated cholesterol efflux, caused a strong increase of prebeta-1 HDL, and drastically changed the distribution of HDL subpopulations. Overall, the results are in line with the hypothesis that MDCO-216 fuses with small alpha-migrating HDL particles forming larger particles containing both apoA-I WT and ApoA-I Milano, meanwhile liberating the endogenous wild-type apoA-I which enriches prebeta-1 HDL subpopulation.

## 1. Introduction

Repeated intravenous administration of recombinant ApoA-I Milano complexed with POPC (previously known as ETC-216) was previously shown to reduce atherosclerotic plaque burden in experimental animals [1, 2] and in man [3]. After improvement of the manufacturing process development of this product (now named MDCO-216) has been resumed by The Medicines Company. We recently reported that infusion of MDCO-216 to cynomolgus monkeys led to rapid and drastic changes in lipoprotein levels and brisk stimulation of global and ABCA1-mediated serum cholesterol efflux capacity after 21 administrations every second day [4].

In the present work we demonstrate that the effect on serum cholesterol efflux capacity could already be obtained when MDCO-216 was incubated with human serum in vitro. We have also investigated the effects of the incubation of MDCO-216 with plasma on the HDL subpopulation profile and HDL prebeta-1 concentration, using a specific ELISA as well as nondenaturing 2D electrophoresis, and on HDL size and particle concentrations by ^1^H-NMR.

## 2. Materials and Methods

MDCO-216 is a complex of highly purified dimeric recombinant ApoA-I Milano and palmitoyl-oleoyl-phosphatidylcholine (POPC). Production of the recombinant protein in* E*.* coli* and its purification have been described [5]. Complexation with POPC was performed using a high-pressure homogenization procedure. The final product (stock solution) contained 13 mg/mL protein, 14 mg/mL POPC, 1.3 mg/mL Di-Na. hydrogen phosphate heptahydrate, 0.178 mg/mL Na. dihydrogen phosphate dehydrate, 62 mg/mL sucrose, and 8.2 mg/mL mannitol, pH 7.5.

### 2.1. Cholesterol Efflux Capacity

MDCO-216 was preincubated with human serum or 4% human serum albumin in concentrations between 100 and 1000 *μ*g/mL (based on ApoA-I Milano protein) at 0 or 37°C. Global and ABCA1-mediated efflux capacity from J774 cells was assessed as described before [4] using 2% of these preincubation mixtures.

### 2.2. Prebeta-1 HDL Quantitation by ELISA

Prebeta-1 HDL in human plasma or in preincubated plasma/MDCO-216 mixtures (see below) was measured at Pacific Biomarkers, using a commercial prebeta-1 HDL ELISA kit from Sekisui Medical Co. LTD. Samples taken from the preincubations were diluted 50-fold with 50% sucrose and kept at −70°C until analysis. The prebeta-1 HDL assay has been described in detail [6].

Preincubation of MDCO-216 with human plasma was done as follows.

Four solutions of drug were prepared:

- Vehicle: 6% sucrose and 1% mannitol in phosphate buffer, pH 7.4;
- Solution High (10 mg/mL): 200 *μ*L MDCO-216 stock + 60 *μ*L Vehicle;
- Solution Medium (3 mg/mL): 100 *μ*L MDCO-216 stock + 330 *μ*L Vehicle;
- Solution Low (1 mg/mL): 50 *μ*L Solution High + 450 *μ*L Vehicle.

All four solutions were spiked into plasma samples of 3 separate donors (50 *μ*L spike + 450 *μ*L plasma) or into Vehicle to obtain final concentration of MDCO-216 at 0, 100, 300, and 1000 *μ*g/mL. 250 *μ*L of each mixture was placed on ice and 250 *μ*L was incubated at 37°C in a water bath.

At the end of the incubation period, all samples, with and without added MDCO-216, either at 0°C or 37°C, were stabilized by diluting 1 : 21 with 50% sucrose (50 *μ*L sample + 1 mL sucrose) and stored at −70°C until analysis.

### 2.3. 2D Electrophoresis

Similar incubations of human serum at 0°C or 37°C were done with or without MDCO-216 and submitted to 2D electrophoresis as described in [7]. Gels were electrotransferred to nitrocellulose membranes and then reacted with either polyclonal goat antihuman apoA-I antibody recognizing both wild-type apoA-I and ApoA-I Milano or with a monoclonal antibody (17F3) specific for ApoA-I Milano, obtained from Mabtech, Nacka, Sweden.

### 2.4. ^1^H NMR

Changes in lipoprotein particle concentrations as a function of time after mixing MDCO-216 with serum were determined by NMR spectroscopy at LipoScience, Inc. (Raleigh, NC) using the modification of the published procedure for analyzing serum [8, 9]. After rapid mixing of a serum specimen with MDCO-216 (430 *μ*g/mL final concentration) and transferring to the flow cell of a 400 MHz NMR analyzer (Bruker Biospin) maintained at 47°C, NMR spectra (130 sec acquisition time) were obtained every 4 minutes over a 1-hour time period. The midpoint of the first spectrum was 2.8 min after mixing, 6.8 min for the second, 10.8 for the third, and so forth. Separate spectra of MDCO-216 and serum at the same concentrations as in the mixture were also acquired. Digital addition of these 2 spectra created an artificial “time zero” (0 min) mixture spectrum to enable assessment of the lipoprotein composition prior to any particle remodeling induced by MDCO-216.

Lipoprotein subclass particle concentrations were calculated from the amplitudes of the spectroscopically distinct lipid methyl group signals emitted by each subclass, derived by deconvolution of the plasma methyl signal envelope at ~0.8 ppm [8, 9]. This computational approach assumes that “the whole is the sum of its parts,” with the parts in the case of human serum comprising reference signals from all of the different-size spherical lipoprotein particles, ranging from the largest chylomicron to the smallest HDL subclass [8]. To successfully analyze mixtures of serum and MDCO-216, we expanded the deconvolution model to include an additional reference signal from MDCO-216 to account for its spectral contribution to the methyl signal envelope of the mixture. Since MDCO-216 is believed to be discoidal rather than spherical in structure, its NMR signal frequency cannot be used to infer its particle diameter nor can its deconvolution-derived signal amplitude be used to calculate its absolute concentration. To monitor and display (in Figure 4) the time-dependent changes in MDCO-216 relative concentration following its addition to serum, we arbitrarily set its “time zero” concentration to 10 *μ*mol/L. Diameter ranges of the HDL particle subclasses quantified by NMR are as follows: large HDL-P 9.4–14 nm, medium HDL-P 8.2–9.4 nm, and small HDL-P 7.3–8.2 nm.

## 3. Results

### 3.1. Preincubation of MDCO-216 with Human Serum Potentiates ABCA1-Mediated Efflux but Not with Basal Efflux from J774 Cells

**Figure 1:**
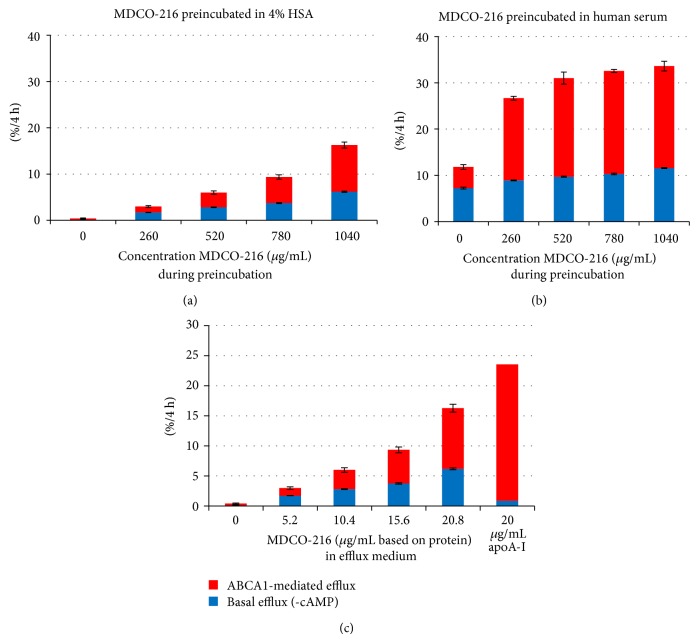
(a) and (b) Effect of MDCO-216 preincubated with 4% HSA (a) or with human serum (b) on global and ABCA1-mediated cholesterol efflux from J774 macrophages. Blue: basal efflux (efflux from cells not pretreated with cAMP), red bars: ABCA1-mediated efflux (additional efflux due to pretreatment of cells with cAMP). (c) The same data of (a) giving concentration of MDCO-216 (as protein) in the incubation medium of the efflux assay, compared with efflux induced by 20 *μ*g/mL pure human wild-type apoA-I (uncomplexed) in the same run.

MDCO-216-HSA and MDCO-216-serum mixtures were compared for their capacity to efflux cholesterol from J774 cells. As shown in Figures 1(a) and 1(c), MDCO-216 preincubated with 4% HSA stimulated basal efflux and ABCA1-mediated cholesterol efflux in a concentration-dependent manner. At a concentration of 20.8 *μ*g/mL (based on ApoA-I Milano protein) of MDCO-216 in the efflux medium, ABCA1-mediated efflux was about half of that obtained by 20 *μ*g/mL free (uncomplexed) human wild-type apoA-I (Figure 1(c)).

Preincubation of serum with MDCO-216 at 37°C led to a concentration-dependent synergistic increase in ABCA1-mediated efflux (compare Figure 1(a) with Figure 1(b)), which means that the measured efflux of the serum-MDCO-216 combination exceeded the sum of the effluxes measured for the individual components. The synergistic effect was also clearly time-dependent, with about half of the increase seen after 10 minutes of preincubation at 37°C (not shown). After 60 min preincubation with serum, maximal stimulation of ABCA1 efflux was reached at 0.5 mg/mL MDCO-216, whereas MDCO-216 preincubated with HSA did not reach maximum at 1 mg/mL. The synergistic effect was seen in similar experiments with other sera having higher ABCA1-mediated efflux capacities (not shown).

Preincubation of serum with MDCO-216 also increased basal (cAMP independent) efflux, but the combination did not reach the sum of the individual components.

### 3.2. Preincubation of Human Plasma with MDCO-216 Protects against Loss of Endogenous Prebeta-1 HDL and Generates New Prebeta-1 HDL as Measured by ELISA and 2D Electrophoresis

As shown in Table 1(a), MDCO-216 itself was detected to some degree by the prebeta-1 HDL ELISA. After incubation of the Low, Medium, and High Solutions at 0°C, the measured amount was about 12% of the real amount of MDCO-216 protein present, whereas after incubation at 37°C this was reduced to about 5%.

Table 1(b) shows that preincubation of plasma alone at 37°C caused loss of nearly 80% of the prebeta-1-HDL ELISA compared to plasma kept at 0°C. After preincubation of plasma with 100, 300, and 1000 *μ*g/mL MDCO-216 at 0°C the amounts of prebeta-1-HDL ELISA ascribable to plasma were 115, 125, and 117%, respectively, of the amounts in plasma incubated with Vehicle. However, after incubation of these mixtures at 37°C amounts of prebeta-1-HDL ELISA increased to 733%, 1220, and 2888%, respectively, of plasma incubated with Vehicle. This suggests that during preincubation at 37°C the added MDC0-216 led to generation of new prebeta-1 HDL in a dose-dependent manner.

**Figure 2:**
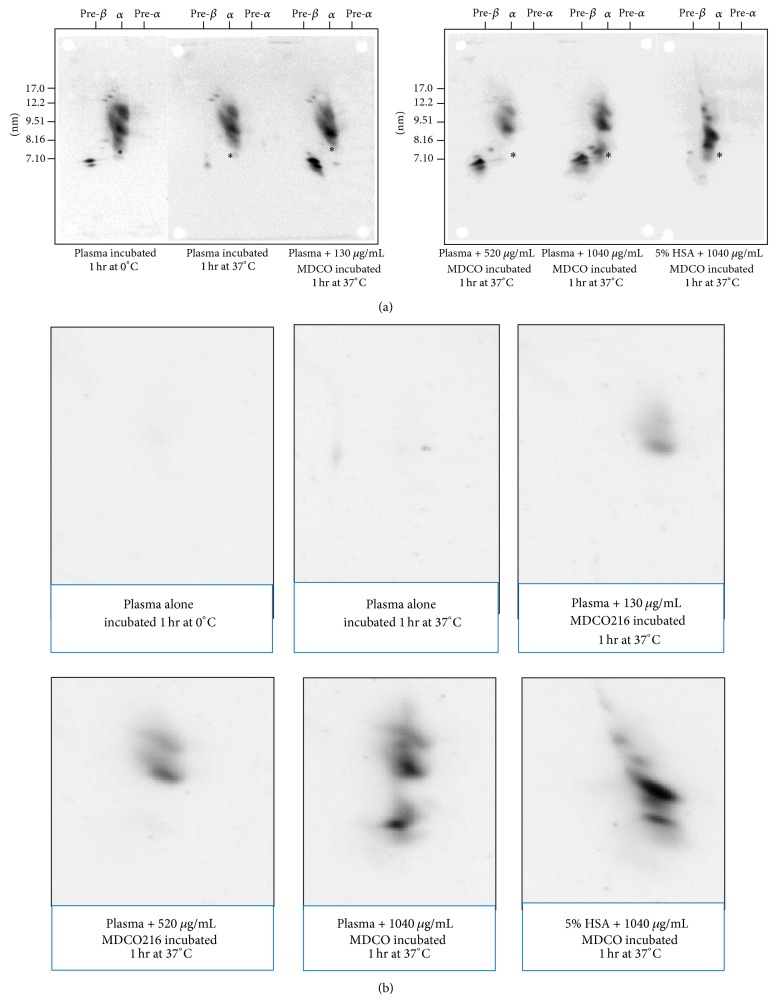
Effect of preincubation of human plasma alone or with MDCO-216 at 37°C. (a) After 2D electrophoresis, the blots were reacted polyclonal Ab against human apoA-I (also reacting with ApoA-I Milano). Asterisk denotes position of albumin. (b) 2D electrophoresis of the same samples of (a), with the blots now reacted with MAb 17F3 specific for ApoA-I Milano.

After similar incubations, samples were subjected to 2D electrophoresis. As shown in Figure 2(a), incubation of serum alone at 37°C leads to a marked loss of prebeta-1 HDL and also some loss of small (alpha-4 and alpha-3) HDL (compare left with middle blot in left panel). MDCO-216 alone showed several alpha-mobility particles with sizes between 8 and 9.5 nm, but no particles with prebeta-1 mobility (right-hand blot in right panel). After incubation of serum spiked with increasing concentrations MDCO-216, there was a prominent increase of prebeta-1 concentration, accompanied with a decrease/disappearance of small alpha-3 and alpha-4 particles and a relative increase in large alpha-1 and alpha-2 particles. Cross immunoprobing the membranes with the ApoA-I Milano-specific antibody (17F3) (shown in Figure 2(b)) indicated that apoA-I Milano was present in alpha-2 and alpha-1 particles. At higher concentrations ApoA-I Milano was also observed in particles similar to alpha-4. However, no ApoA-I Milano was detected in prebeta-1 HDL.

### 3.3. Remodeling of Serum HDL upon Incubation with MDCO-216 Assessed by ^1^H-NMR

**Figure 3:**
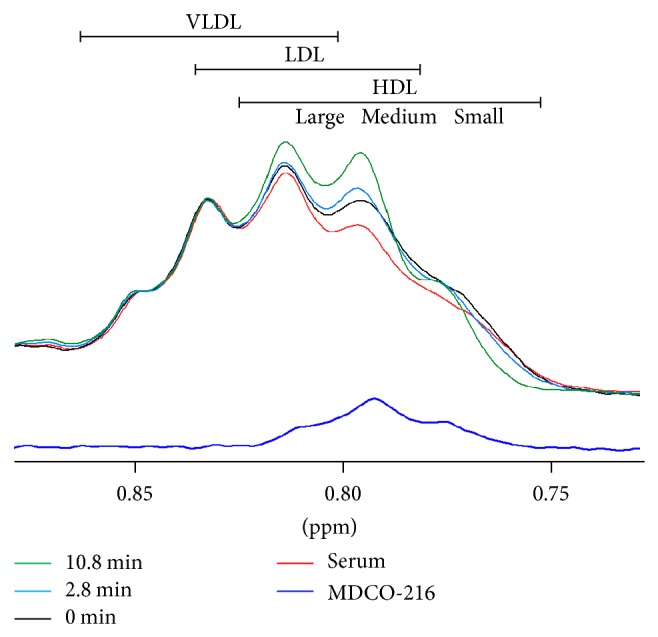
Changes in the ^1^NMR lipid methyl signal envelope brought about by incubation of MDCO-216 with serum at 47°C. The signals shown are from MDCO-216 (dark blue) and the serum specimen (red) before they were mixed together. The digital sum of these 2 signals (black) represents the mixture at “0 min” before physical mixing occurs, and the blue and green curves are the signals observed after 2.8 and 10.8 minutes of incubation, respectively. Also the positions in the serum spectrum of the methyl signals from the subclasses of VLDL, LDL, and HDL, with larger subclasses giving rise to signals further to the left [8], are shown at the top.

The phospholipid in MDCO-216 gave rise to a methyl NMR signal centered at 0.79 ppm (Figure 3). A signal of this frequency would correspond to that from a medium-size spherical HDL particle [8], but because the structure of MDCO-216 may not be spherical, it is not currently possible to deduce its size or structure from its NMR characteristics. Also in Figure 3 the methyl signal envelopes of the serum sample (red), the artificial “0 min” mixture created by digital addition of the serum and MDCO-216 signals (black), and the signals resulting from incubation of MDCO-216 and serum for 2.8 min (blue) and 10.8 min (green) are shown. Qualitative inspection of these curves indicates a time-dependent reduction of signal amplitude in the region coming from by small HDL particles (ca. 0.77 ppm) and a corresponding increase in signal coming from medium- and large-size HDL subclasses (ca. 0.79–0.82 ppm). Prebeta-1 HDL particles do not appreciably contribute signal to this region of the NMR spectrum due to their very low lipid content.

**Figure 4:**
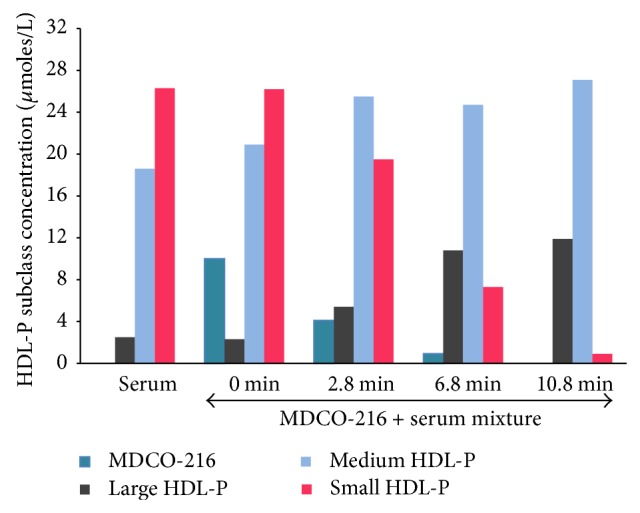
Changes in MDCO-216 and HDL subclass concentrations brought about by incubation of MDCO-216 with serum at 47°C. HDL subclass concentrations (*μ*mol/L) were derived by deconvolution analysis using a model that included MDCO-216. Only relative concentrations of MDCO-216 are given, with the starting concentration in the 0 min digital mixture set arbitrarily to 10 *μ*mol/L for graphical display purposes.

The quantitative assessment via deconvolution analysis of changes in MDCO-216 and HDL particle subclass concentrations brought about by addition of MDCO-216 to serum is shown in Figure 4. Not shown are data for the VLDL and LDL subclasses which were unaffected by addition of MDCO-216, nor for the HDL subclasses at longer time points since further changes beyond 10.8 min were not observed. The signal from MDCO-216 decreased rapidly over time and was not detectable in the 10.8 min spectrum and beyond. Over the same short time interval, the concentration of small HDL particles fell >95% while there was a concomitant increase in medium-size HDL and a substantial 5-fold increase in the concentration of large HDL particles. These subclass changes led to a net decrease in total HDL particle concentration from 47 to 40 *μ*mol/L.

## 4. Discussion

The data presented here show that incubation of plasma or serum with MDCO-216 in vitro causes a strong increase in ABCA1-mediated cholesterol efflux capacity, concomitant with increased average HDL size and generation of new prebeta-1 HDL containing only wild-type apoA-I.

Recently, very similar changes were reported for incubation of human serum with CSL-112 [10]. CSL-112 consists of two molecules wild-type apoA-I complexed with 110 molecules soybean lecithin. A strong increase in ABCA1-mediated efflux was observed after incubation of CSL-112 with human serum at 37°C* in vitro*. Incubation of 1 mg/mL CSL-112 with serum at 37°C was described to strongly increase prebeta-1 HDL concentration, whereas CSL-112 was hardly detectable with the prebeta-1-HDL ELISA assay. The increase reported by these authors was very similar to that reported here (Table 1) after incubating serum with 1 mg/mL MDCO-216. The authors report that upon one-dimensional nondenaturing gradient PAGE this incubation led to marked generation of particles with the same size (about 5.6 nm) as seen after incubation of serum with free apoA-I. In our case, native 2D electrophoresis demonstrated a marked increase in particles with prebeta-1 mobility and apparent size of 7.2 nm. Miyazaki et al. recently provided evidence that the particles detected as prebeta-1 HDL upon 2D electrophoresis and by the Sekisui ELISA actually consist of lipid-free apoA-I [11].

Our ^1^H-NMR measurements showed rapid conversion of MDCO-216 and small-sized HDL into larger-sized HDL particles, with a concomitant decrease in the total HDL particle concentration. There was no evidence from the deconvolution analysis that MDCO-216 persisted in its native structural state beyond about 10 minutes of incubation with serum, but we cannot rule out the possibility that it may have been transformed into a different structure(s) with unknown NMR properties. The validity of the NMR deconvolution approach to deduce HDL subclass changes, caused by addition of MDCO-216, is supported both by the observed agreement with the HDL changes seen by 2D electrophoresis and by numerous reports in the literature of agreement between NMR-assessed lipoprotein changes and those assessed independently using various separation techniques [12–16]. The latter also applies for the lack of effect on LDL and VLDL size and particle numbers inferred by NMR deconvolution.

Taken together, the data reported here support the hypothesis that upon incubation with plasma or serum, MDCO-216 fuses with small endogenous alpha-migrating HDL particles to produce larger HDL particles containing both apoA-I WT and ApoA-I Milano. As a side effect of this fusion, free apoA-I WT is liberated which is detected as prebeta-1 HDL. Similar results have been described for an apoA-I mimetic peptibody after intravenous administration in mice [17]. The question remains whether the increase in ABCA1-mediated efflux is brought about solely by these newly-formed prebeta-1 HDL particles or whether the altered (larger) HDL particles now containing ApoA-I Milano also contribute to the increased cholesterol efflux capacity of serum.

## Figures and Tables

**(a):** 

Solution spiked	Vehicle	Low	Medium	High

Final MDCO-216 concentration (*μ*g/mL protein):	0	100	300	1000

		Prebeta-1 HDL in *μ*g/mL (% of added)		
Incubated at 0°C	0	12 (12%)	44 (15%)	118 (12%)
Incubated at 37°C	0	5 (5%)	13 (4%)	52 (5%)

**(b):** 

Incubation temp.	1 h 0°C			

Solution spiked	Vehicle	Low	Med	High

Final MDCO-216 concentration (*μ*g/mL protein):	0	100	300	1000

		Prebeta-1 HDL net due to plasma^*^		
Spiked in plasma 1	62	75	101	105
Spiked in plasma 2	126	126	131	121
Spiked in plasma 3	112	144	143	125
Spiked in plasma average	100	115	125	117
% of plasma spiked with vehicle^**^		115%	125%	117%

Incubation temp.	1 h 37°C			

Solution spiked	Vehicle	Low	Med	High

^*^Final MDCO-216 concentration (*μ*g/mL protein):	0	100	300	1000

		Prebeta-1 HDL net due to plasma^*^		
Spiked in plasma 1	5	77	162	379
Spiked in plasma 2	12	89	153	375
Spiked in plasma 3	23	127	173	401
Spiked in plasma average	13	98	163	385
% of plasma spiked with vehicle^**^		733%	1220%	2888%

^*^Value for MDCO-216 spiked in plasma minus value for MDCO-216 spiked in Vehicle (Table 1(a)) in *μ*g/mL. ^**^(value for plasma plus MDCO-216 minus value for MDCO-216 in vehicle)/value for plasma spiked with vehicle.
